# The impact of leprosy on the mental wellbeing of leprosy-affected persons and their family members – a systematic review

**DOI:** 10.1017/gmh.2020.3

**Published:** 2020-06-09

**Authors:** PMW Somar, MM Waltz, WH van Brakel

**Affiliations:** 1 VU University Amsterdam, Amsterdam, Netherlands; 2 NLR, Amsterdam, Netherlands

**Keywords:** Anxiety, depression, leprosy, mental health, neglected tropical disease

## Abstract

Leprosy has long-term consequences related to impairment and stigma. This includes a major impact on mental health. This study aims to consolidate current evidence regarding the mental health impact of leprosy on affected persons and their family members. In addition, determinants influencing mental health outcomes among leprosy-affected persons and effective interventions are examined. A keyword-based search was conducted in PubMed, Web of Science, Scopus, PsycINFO, Infolep and InfoNTD; additional literature was also considered. Articles presenting primary data involving leprosy-affected persons or their family members experiencing mental conditions were included. Independent extraction of articles was executed using predefined data fields. Articles were sorted according to relevance. In total, 65 studies were included in this systematic review. Multiple psychiatric morbidities have been identified among leprosy-affected persons, including depression, anxiety disorders and suicide (attempts). Additional factors were found that may impact mental health. Moreover, studies found that demographic factors, lifestyle and disease-specific factors and stigma and discrimination impact mental health. Depressive symptoms and low self-esteem were identified among children of leprosy-affected persons. In addition, interventions were identified that could improve the mental wellbeing of leprosy patients. Depressive disorders and anxiety disorders were found to be very common among persons affected by leprosy. Feelings such as fear, shame and low self-esteem are also experienced by those affected, and their children. Further research is necessary to ensure that mental health impact is included when determining the burden of disease for leprosy, and to relieve this burden.

## Introduction

Leprosy, a neglected tropical disease (NTD) mainly characterised by skin lesions and damage to peripheral nerves, is estimated to have a burden of disease of around 21100 disability-adjusted life years (DALYs) (Kyu *et al*., 2018). However, until now, quantification of the burden of leprosy has taken a very narrow view, including only the number of new cases in a given year and the proportion among these that have visible – so-called ‘grade 2’ – disabilities. Chronic consequences of leprosy, such as the negative impact on social participation and mental health, have not been taken into account due to data scarcity and the lack of standardised data collection (Jamison *et al*., 2006; Hotez *et al*., 2014). In addition, leprosy develops progressively at the onset, but gradually over time. Quantifying the burden at one particular time is therefore not representative of the full disease course (Richardus, 2013). Moreover, leprosy does not only influence the lives of (former) patients, but also the lives of their direct contacts, such as family members, friends and people in their community. The disability rates that are used to calculate DALYs represent societal preferences for various health states, rather than experienced health of the persons coping with impairment and disability (Hotez *et al*., 2014). Taking the experienced health into account when calculating the burden of leprosy is essential, since leprosy can be demonstrated to have a significant impact on social participation and mental health in addition to causing physical impairments (Bainson and Van den Borne, 1998).

Bainson and Van den Borne (1998) described the mental health effects of having leprosy. They found that leprosy follows both a biomedical and a social course. In the biomedical course, impairments caused by leprosy lead to emotional reactions and negative behaviours. In the social course, persons with leprosy encounter social barriers that lead to disability, emotional reactions and negative behaviours. Both the biomedical and the social trajectory can contribute to unemployment, economic and physical dependence and difficulty with social integration. This can lead to dehabilitation and consequently to destitution (Bainson and Van den Borne, 1998). Litt *et al*. (2012) further examined the relationship between NTDs, including leprosy, and mental health conditions. They found that the consequences of NTDs include stigma, social exclusion, reduced access to healthcare services, lack of educational and employment opportunities, restriction of rights, increased disability and early mortality. Each of these consequences may result in poor mental wellbeing by increasing feelings and behaviours such as sadness, hopelessness and social withdrawal. Poor mental wellbeing and other consequences of an NTD can both contribute to the development of mental health conditions, such as anxiety and depression (Litt *et al*., 2012).

The psychological and societal consequences of leprosy have been widely researched, in particular in relation to disability and stigma (Cross and Choudhary, 2005; Sermrittirong and Van Brakel, 2014). However, no systematic review had been done to consolidate the evidence regarding the specific impact of leprosy on mental health, including mental health conditions. Additionally, little is known about the impact of leprosy on family members who are indirectly affected. Therefore, the aim of this study is to review existing studies regarding mental health and leprosy in order to make an inventory of the ways in which leprosy impacts the mental health of people affected and their family members.

## Methods

A systematic search was conducted in six online databases: PubMed, Web of Science, Scopus, PsycINFO, Infolep and InfoNTD to identify relevant studies regarding the impact of leprosy on the mental health of those affected and their family members. A generic syntax was set up consisting of keywords found in articles that were used for orientation regarding the subject and articles suggested by leprosy experts. The keywords were: leprosy, Hansen's disease, NTD, mental disease, mental condition, mental health, psychologic, psychiatric, depression, anxiety and suicide. Variations of these keywords were also used to devise syntaxes for the various databases (see Table 1). In addition, other relevant studies and literature were found via Google Scholar and the ‘snowball method’ (bibliography screening of relevant articles for other relevant articles).

**Table 1.** Syntaxes for search

Search engine	Syntax
PubMed	((((Leprosy[Title] OR Hansen's disease [Title] OR (NTD [Title] AND leprosy [text word])) AND (mental health[Title/Abstract] OR psychologic* [Title/Abstract] OR psycho* [Title/Abstract] OR mental* [Title/Abstract] OR psychiatric* [Title/Abstract] OR suici* [Title/Abstract] OR (depression NOT cell NOT macrophage NOT immun* NOT lymphocyt* [Title/Abstract]) OR depressive [Title/Abstract] OR anxi*[Title/Abstract]))))
Web of Science	(#1 OR #2) AND #3 #1: TI = (Leprosy OR Hansen's disease) #2: TI = NTD AND TS = leprosy #3: TS = [mental health OR psychologic* OR psycho* OR mental* OR psychiatric* OR suici* OR (depression NOT cell NOT macrophage NOT immun* NOT lymphocyt*) OR depressive OR anxi*]
Scopus	TITLE (leprosy OR ‘Hansen's disease’) OR [TITLE (‘neglected tropical disease’) AND TITLE-ABS-KEY (leprosy)] AND TITLE-ABS [(‘mental health’) OR psychologic* OR psycho* OR mental* OR psychiatric* OR suici* OR depressive OR anxi* OR ‘depression AND NOT macrophage AND NOT immun* AND NOT lymphocyte AND NOT antigen AND NOT cell’]
PsychINFO	TI [(leprosy or ‘Hansen's disease’)] AND AB ((‘mental health’ or psychologic* or psycho* or mental* or psychiatric* or suici* or (depression not cell not macrophage not immun* not lymphocyt*) or depressive or anxi*))
Infolep	Leprosy or Hansen's disease + (‘mental health’ ‘depression’ suicid psych anxi mental)
InfoNTD	Leprosy or Hansen's disease + (‘mental health’ ‘depression’ suicid psych anxi mental)

Studies were excluded from the search if they had a primarily biomedical character, were reviews or meta-analyses, were written in a language other than English or Dutch, portrayed the perspective of people other than persons affected by leprosy or their family members or were obviously irrelevant in any other way, for instance, having content with no relation to mental health. The final search was performed on 8 February 2018.

All articles identified in the search were exported in a reference manager (EndNote). Duplicates were automatically removed. The next step consisted of title and abstract screening. Irrelevant articles were excluded. Next, screening of full text articles was done. Both the first and second screening were performed by one author (PS) and supervised by two other authors (WvB, MW). Uncertainties regarding inclusion or relevance of data were resolved together with the second authors. The title, abstract and full-text screening were executed in Covidence, a web-based software platform that streamlines the production of systematic reviews. Inclusion and exclusion criteria were used to guide the screening process (see Table 2).

**Table 2.** Inclusion and exclusion criteria

Inclusion criteria	Exclusion criteria
*Study content*: studies including leprosy patients comorbid with one or multiple psychiatric morbidities, studies including family members of leprosy patients, studies directed at interventions for improving mental health in leprosy-affected persons or their family members	*Study content*: studies that did not include primary data on persons affected by leprosy or their family members and mental health, and biomedical studies, or other irrelevant articles (wrong subject)
	*Type of study*: (systematic) reviews, and meta-analysis
	*Language*: articles that were not in English or Dutch

Data extraction was done in Excel. Relevant information including study characteristics, patient characteristics, morbidity specifics and outcomes was noted. The articles were labelled based on the relevance. The relevance of the articles was determined based on several characteristics (Table 3). Specific conditions, studies and other topics and themes were noted and results were combined, described and discussed based on similarities and discrepancies between these. Ranking the articles provided a critical view on how to report the results and what to report. The ranking was performed by PS and supervised by MW.

**Table 3.** Criteria of relevant articles

	Level of relevance		
	3	2	1
Study design	All, except for theses, reports, editorials	All	
Main topic	Leprosy and mental health impact	Leprosy and mental health impact, stigma, discrimination, negative feelings	
Sample size	Large	Small	
Outcomes	Measured		Not-measured
Mental health conditions	Specific conditions		Psychiatric morbidity in general or other information regarding mental health and leprosy; personal experience regarding the mental impact of living with leprosy

## Results

**Fig. 1.**
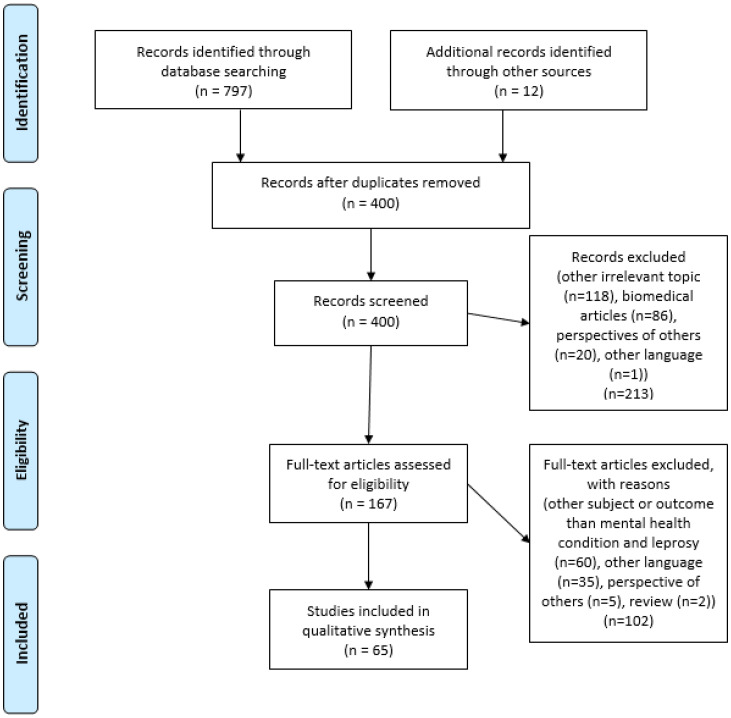
Results in PRISMA flowchart.

In total, 797 articles were found (see Fig. 1). In addition, 12 articles were found via Google Scholar or the ‘snowball method’ by using backwards reference searching and added to the study. Out of the 797 articles, 409 were removed as they were marked as duplicates. After screening titles and abstracts, 213 studies were discarded based on the exclusion criteria: biomedical articles that described cellular processes of mental health or leprosy (*n* = 86), because they were otherwise irrelevant (*n* = 118), represented perspectives of others than leprosy-affected or their family members (*n* = 20) or articles in another language (*n* = 1). In addition, 20 duplicates were removed. A total of 167 studies were included for full text screening. Based on the above exclusion criteria, a further 102 articles were discarded at this stage: other subject or outcome than mental health condition and leprosy (*n* = 60), other language (*n* = 35), perspective of others (*n* = 5), review (*n* = 2). In the end, we included an analysis of 65 relevant articles regarding leprosy and mental health. Of these, 11 of 65 articles were studies describing interventions. Out of 65 studies, 62 concerned leprosy-affected persons, while three studies concerned the family members of the affected persons. The studies included and their important characteristics are presented in Table 4.

**Table 4.** Articles included in review *uploaded separately*

Author(s) (year)	Type of study	Country	Study population	Control group(s)	During or after treatment	Type of psychological impact or mental health condition	Measurements used	Results (prevalence in % of total study population of leprosy-affected persons)	Results (prevalence in % in control group(s))	Level of relevance
Anjum *et al*. (2017)	Cross-sectional	India	54 leprosy-affected persons from a rehabilitation home		Both	Self-discrimination, fear, shame	Pre-tested semi structured questionnaire	Self-discrimination (56%), fear (43%), shame (35%)		2
Antony and Broota (1991)	Case control	India	50 children of leprosy-affected persons	30 children of healthy persons	–	Negative self-concept	Interviews	76%	46%	2
Arole *et al*. (2002)	Cross-sectional	India	24 leprosy-affected persons from vertical and horizontal villages		Both	Low self-esteem	Stigma measurement scale with open-ended questions, focus group discussion and participatory rural appraisal	10%		2
Bainson and Van der Borne (1998)	Review	–	–		–	Low self-esteem, low quality of life	–	–		1
Bakare *et al*. (2015)	Cross-sectional	Nigeria	235 persons from a leprosy camp		Both	Anxiety, depression	General Health Questionnaire (GHQ-28) and the Composite International Diagnostic Interview (CIDI)	Moderate depressive episode (14%), severe depressive episode (6%), GAD (19%), mixed anxiety and depressive disorder (9%)		3
Beise *et al*. (2012)	Thesis	Indonesia	158 persons with and without leprosy			Stress, low self-esteem	–	–		1
Bharath *et al*. (2001)	Cross-sectional	India	30 patients		During treatment	Psychiatric morbidity	GHQ-12	–		1
Bharath *et al*. (1997)	Cross-sectional	India	246 patients from out-patient clinic	63 patients with psoriasis	During treatment	Depression, suicide, anxiety	GHQ-12	Depression (12%), anxiety (8%), suicidal ideas (4%), suicide attempts (2%)	Depression (46%), anxiety (30%), suicidal ideas (21%), suicide attempts (14%)	3
Bhatia *et al*. (2006)	Case control	India	90 leprosy-affected persons from a leprosy clinic	40 patients suffering from acute skin problem		Anxiety	GHQ-12 and Disability Assessment Questionnaire (DAQ)	Generalised anxiety disorder (28%), mixed anxiety and depressive disorder (13%), psychosexual disorder (2%), delusional parasitosis (1%)	Generalised anxiety disorder (5%), mixed anxiety and depressive disorder (2.5%), psychosexual disorder and delusional parasitosis (0%)	3
Rocha-Leite *et al*. (2015)	Case control	Brazil	126 outpatients	52 (out of 126) leprosy patients without neuropathy and without perception of social exclusion	During treatment	Anxiety, depression	Mini International Neuropsychiatric Interview (M.I.N.I. PLUS) and Short Form Health Survey (SF-36)	Anxiety (40%), depression (27%)	Unknown	3
Brouwers *et al*. (2011)	Cross sectional	Nepal	100 leprosy-affected persons			Low quality of life	World Health Organisation Quality of Life (WHOQOL) assessment, Jacoby Scale, Participation Scale and Green Pastures Activity Scale (GPAS)	–		2
Cazenavette (1927)	Review	United States	–		During treatment	Depression, psychosis, dementia, melancholia	–	–		1
Chatterjee *et al*. (1989)	Cross sectional	India	17 in-patients and 16 out-patients	17 pulmonary tuberculosis patients and 19 patients with chronic ear, nose and throat complaints	During treatment	depression, suicidal thoughts	Standard psychiatric interview, the attitude questionnaire and the GHQ-(?)	Depression (100%), suicidal thoughts (33%)	Unknown	2
Chauhan and Dahr(1981)	Cross sectional	India	11 patients (11–18 years)		During treatment	Anxiety	Leopold Bellak's Children's Apperception Test (CAT)	–		1
Costa *et al*. (2012)	Cross sectional	Brazil	120 patients		During treatment	Negative feelings including bad mood, desperation, anxiety and depression	WHOQOL	86%		2
Cunha *et al*. (2015)	Cross sectional	Brazil	130 patients		During treatment	Common mental disease, fear, low self esteem	SRQ-20	Common mental disease (32%), real fear (32%), phantasmal fear (22%), low self-esteem (29%)		3
Dadun *et al*. (2016)	Exploratory	Indonesia	53 leprosy-affected persons		Both	Low self-esteem, anxiety, shame, loneliness, anger, depression, hopelessness	In-depth interviews and focus group discussions	–		1
Damte *et al*. (2011)	Cross sectional	Ethiopia	269 outpatients		During treatment	Mental distress	Self-Reporting Questionnaire (SRQ-20)	31%		3
Enwereji (2015)	Cross sectional	Nigeria	33 discharged leprosy-affected persons		After treatment	Depression, loneliness	Nottingham's life satisfaction index and Beck's inventory on depression indices	Loneliness (100%), depression (0%)		3
Erinfolami and Adeyemi (2009)	Case control	Nigeria	88 patients	88 patients with tinea versicolor (TV) and 88 healthy persons (HP)	During treatment	Anxiety disorders, depression, schizophrenia	GHQ-30	Depression (33%), anxiety disorders (22%), schizophrenia (1%)	Depression (TV 7% and HP 8%), anxiety disorders (TV 10% and HP 7%), schizophrenia (TV 1% and HP 0%)	3
Garbin *et al*. (2015)	Cross sectional	Brazil	94 patients		During treatment	Depressive and sad feelings, low quality of life, fear, shame	Structured questionnaire	Fear of disease sequelae (93%), depression (67%), fear of telling family about disease (52%), low quality of life (46%), depression and sadness (40%), fear of dying (28%)		3
George *et al*. (2013)	Quasi-experimental design	India	40 female patients		During treatment	Anxiety	Nursing assessment and Hamilton Anxiety Rating Scale	Before nursing intervention: moderate or severe anxiety (80%) After nursing intervention: moderate or severe anxiety (5%)		3
Hiletework (2016)	Cross sectional	Ethiopia	20 female patients		During treatment	Loneliness, inferiority, low self-esteem, depression, aggressiveness, shame	In-depth interview, Key Informant Interview (KII), Focus Group Discussion (FGD) and personal observation	Loneliness (25%), inferiority (15%)		2
Hossain *et al*. (2016)	Cross sectional	Bangladesh	92 patients			Low self-esteem, loneliness	Semi-structured questionnaire	30% of the men and 14% of the women felt no respect from colleagues, 27% of the men and 8% of the women felt avoided at work		2
Jindal *et al*. (2013)	Cross sectional	India	133 leprosy-affected persons in leprosy homes		After treatment	Depression, anxiety, schizophrenia	GHQ-12, WHO disability scale, ICD-10	Dysthymia (36%), moderate depressive disorder (15%), GAD (11%), mixed anxiety and depressive disorder (3%), schizophrenia (1%)		3
Jopling (1991)	Review					Suicide (attempts), shame, fear	–			1
Joseph and Rao (1999)	Case control	India	50 leprosy-affected persons	50 healthy persons	After treatment	Low quality of life	WHOQOL	79%	85%	1
Kamiya (1962)	Cross sectional	Japan			During treatment	Schizophrenia, depression, anxiety, epilepsy, suicide (attempts)	–	145 cases of psychiatric morbidity in a leper community in a year		2
Kataoko and Nakamura (2005)	Cross sectional	Japan	232 patients		During treatment	Psychological wellbeing	GHQ-12	Significantly higher GHQ-12 scores (lower psychological wellbeing) were identified in men who had fewer social factors, no close friends and no participation in group activities. People with inactive daily lives scored higher GHQ-12 scores		2
Kaur and Ramesh (1994)	Cross sectional	India	50 female patients from leprosy centres		During treatment	Fear, loneliness, anger, sadness, embarrassment	Interviews	70% were easily upset, 62% had a tendency to get angry over trivial matters, 58% felt ashamed or embarrassed by their physical imperfection, 50% feared infecting their families, 48% feared questions about disease, 30% preferred to remain alone		3
Kisivuli *et al*. (2005)	Cross sectional	Kenya	152 leprosy-affected persons			Depression, anxiety	Questionnaire	General anxiety disorder (18%), somatoform disorders (9%), depression (7%)		3
Kumar and Verghese (1980)	Cross sectional	India	494 patients		During treatment	Depression	Mental Health Item sheet and the M-R scale of the Cornell Medical Index	Depressive reaction (8%)		2
Lasry-Levy *et al*. (2011)	Cross sectional	India	101 leprosy-affected persons		After treatment	Psychological morbidity	Thee Douleur Neuropathique 4 and GHQ-12	15%		1
Latheef and Riyaz (2014)	Quasi-experimental design		2 cases			Anxiety, self-esteem	–	S		1
Leekassa *et al*. (2004)	Case control	Ethiopia	471 leprosy-affected persons	315 people with other skin diseases visiting ALERT outpatient clinic	During treatment	Mental distress	Self Reporting Questionnaire (SRQ)	52%	8%	3
Leite and Caldeira (2015)	Quasi-experimental design	Brazil	62 leprosy-affected persons			Depression	The Beck Depression Inventory and WHOQOL-bref	Therapeutic workshops decreased depression from 79% to 47%, and moderate depression from 55% to 18%		3
Mahendra *et al*. (2016)	Cross sectional	India	100 leprosy-affected persons		Both	Mood disorder, anxiety disorder, psychotic disorder	Semi-Structured Self Prepared Pro-forma and ICD-10 Checklist for Mental Disorders	Mood disorder (30%), anxiety disorder (10%), psychotic disorder (2%), hypochondrical disorder (1%), adjustment disorder (1%)		3
Mhasawade (1983)	Quasi-experimental design	India	120 leprosy-affected persons			Depression, anxiety	Taylor's anxiety scale and Beck's scale for depression	Psychotherapy lowered anxiety and depression levels		1
Moura *et al*. (2017)	Cross sectional	Brazil	56 patients		During treatment	Depression	Hamilton depression scale	Severe depression (9%), moderate depression (16%), mild depression (34%)		3
Nagargoje *et al*. (2015)	Cross sectional	India	80 leprosy-affected persons		Both	Depression, anxiety, suicide	socio demographic questionnaire, Duke's GHQ, DSM-5 self rated level 1 cross cutting symptom measure – adult and WHO-QOL-BREF	Depression (28%), anxiety disorders (24%), somatic symptom disorder (19%), suicidal ideation (12%), sleep disorders (6%), substance use disorders (5%)		3
Nishida *et al*. (2006)	Cross sectional	Japan	385 elderly leprosy-affected persons from a leprosarium		Both	Depression, suicide	Mini-mental state examination (MMSE), the Geriatric Depression Scale Short Form (GDS-SF), the Philadelphia Geriatric Center (PGC) morale scale and the Tokyo Metropolitan Institute of Gerontology index (TMIG index)	Depression (13%), 41 cases of suicide since the establishment of leprosarium		2
Parashar and Kumar (2015)	Cross sectional	India	100 adolescent children from leprosy-affected persons			Low self-esteem	WHOQOL and Rosenberg's self-esteem scale	Low self-esteem (60%)		3
Pérez-Hernández *et al*. (2015)	Quasi-experimental design	Mexico	24 leprosy-affected persons			Self-concept (self-esteem, body image, body sensation, conscious self, ideal self, moral self)	‘Viveros 03’ instrument for persons with chronic diseases	The technique of directed imagination significantly increased the adaptation level score of the self-concept mode from 92% to 96%		2
Peters *et al*. (2014)	Cross sectional	Indonesia	53 female leprosy-affected persons		Both	Suicidal thoughts, shame, sadness, low self-esteem, depression	Qualitative semi-structured in-depth interviews	The women who had concealed their illness the most (29%) reported feeling more emotions such as sadness, shame, low self-esteem and depression. Perceived stigma could lead to self-mutilation or suicidal thoughts.		1
Rahmawati and Yuniarti (2017)	Quasi-experimental design	Indonesia	25 leprosy-affected persons			Depression	Beck Depression Inventory	CBT lowered depression rates from 100% to 88%		2
Ramanathan *et al*. (1991)	Quasi-experimental design	India	25 leprosy-affected persons			Depression, anxiety	Beck's depression inventory, Taylor's manifest anxiety scale test, Thematic apperception test (TAT) and Wechsler adult intelligence scale test	Before surgical correction: anxiety (80%), depression (72%) After surgical correction: anxiety (40%), depression (40%)		2
Reis *et al*. (2014)	Cross sectional	Brazil	21 leprosy-affected persons with neuropathic pain		After treatment	Psychological distress, psychiatric morbidity	GHQ-12 and WHOQOL-bref	High psychological distress (76%), low psychological distress (24%), psychiatric morbidity (76%)		2
Rocha-Leite *et al*. (2014)	Cross sectional	Brazil	120 patients		During treatment	Depression, anxiety, suicide, panic disorder, OCD/BDD	Sociodemographic questionnaire and M.I.N.I. PLUS	Depression (31%), panic disorder (16%), agoraphobia (12%), obsessive-compulsive disorder (15%), body dysmorphic disorder (12%), GAD (9%), social phobia (9%), somatoform disorder (6%), risk of suicide (71%)		3
Sanyal *et al*. (2011)	Cross sectional	India	93 patients		During treatment	Psychiatric morbidity	GHQ-60, SCARF social functioning index and a semi-structured proforma	54%		2
Scott (2006)	Cross sectional	South-Africa	10 leprosy-affected persons			Fear, suicidal thoughts	Interviews	There were no suicidal thoughts among study participants. Some feared and experienced stigmatisation and discrimination		1
Shen *et al*. (2011)	Cross sectional	China	524 cases of deceased patients		During treatment	Suicide	–	Suicide was the cause of death in 16% of the cases		3
Sillo *et al*. (2016)	Cross sectional	Brazil	27 leprosy-affected persons		Both	Depression, anger	Semi-structured face-to-face interviews	The combination of experienced stigma and the inability to work due to physical deformities caused depression, anger and self-stigmatisation.		1
Su *et al*. (2011)	Randomised controlled	Taiwan	129 elderly leprosy-affected persons		After treatment	Dementia, depression	The Geriatric Depression Scale – Short Form (GDS-SF), the mini mental state examination (MMSE) and the Clinical Dementia Rating (CDR) scale	Dementia (48%), depression (25%). RGT significantly lowered the depression rates		3
Thilakavathi *et al*. (2015)	Cross sectional	India	155 leprosy-affected persons		Both	Fear	In-depth interviews	Fear or worry to experience stigma (13%)		2
Thwaites *et al*. (2014)	Cross sectional	India	30 leprosy-affected persons		Both	Anxiety	Semi-structured interview	26% [due to leprosy complications (35%), employment concerns (26%), curability (17%), drug side effects (13%) and stigma (9%)]		3
Tsutsumi *et al*. (2004)	Case control	Bangladesh	140 leprosy-affected persons	135 local people without chronic diseases	Both	Depression	Center for Epidemiologic Studies Depression scale (CES-D)	CES-D score of 28	CES-D score of 12	3
Tsutsumi *et al*. (2007)	Case control	Bangladesh	189 leprosy-affected persons	200 persons without leprosy or other chronic diseases		Quality of life	WHOQOL-BREF, self-reporting questionnaire, Barthel index and Perceived Stigma Questionnaire	Leprosy patients had significantly worse total QOL scores as well as lower physical and psychological domain scores than the general population for both genders	*See results of leprosy-affected persons*	2
Utami *et al*. (2017)	Cross sectional	Indonesia	72 patients		During treatment	Depression	Structured questionnaire	Cognitive, psychological and social factors have a significant effect in preventing depression		1
Verghese *et al*. (1971)	Case control	India	20 male patients from sanatorium	15 patients with other chronic diseases, 15 neurotic patients and 15 healthy persons	During treatment	Neuroticism	Psychiatric interview, Eysenck Personality Inventory and Cattel's 16 Personality Factor Inventory	The leprosy group had high neuroticism score, and showed a tendency to have several neurotic traits	The group with other chronic diseases also had a high neuroticism score	2
Verma and Gautam (1994-I)	Cross sectional	India	100 leprosy-affected persons from an ashram and slum areas		Both	Depression, anxiety	GHQ-(?)	Neurotic depression (55%), anxiety neurosis (21%). People with deformities had more often psychiatric morbidity than people without deformities (90% v. 47%). Significant difference for psychiatric morbidity was seen in occupation, marital status, family income and family size		3
Verma and Gautam (1994-II)	Cross sectional	India	100 leprosy-affected persons from an ashram and slum areas		Both	Depression, anxiety	GHQ-(?)	Neurotic depression (55%), anxiety neurosis (21%). Psychiatric morbidity was significantly higher in patients living in slum areas		3
Weiss *et al*. (1992)	Cross sectional	India	56 patients		During treatment (start of treatment)	Depression, anxiety	Explanatory Model Interview Catalogue	Depressive disorder (71%), anxiety disorder (43%)		3
Yamaguchi *et al*. (2013)	Cross sectional	Nepal	102 adolescents with leprosy-affected parents			Depression, health-related quality of life, low self-esteem	Kinder Lebensqualität Fragebogen (KINDLR), the Center for Epidemiological Studies-Depression Scale (CESD) and the Rosenberg Self-esteem Scale (RSES)	Adolescents with leprosy-affected parents had higher levels of depressive symptoms, lower levels of self-esteem, and lower QoL compared with adolescents whose parents were unaffected by leprosy		3
Yirga (2016)	Cross sectional	Ethiopia	22 patients		During treatment	Suicide (attempts), depression, anxiety	–	Almost all participants confirmed that they have experienced a suicide attempt. Anxiety and depression were also very common among participants		1
Zambon Valério Pelizzari *et al*. (2016)	Cross sectional	Brazil	9 leprosy-affected persons		Both	Fear, shame	Semi-structured interviews	Subjects reported feeling ashamed to say to others who possessed the disease, by believing that people might fear the disease or to prevent manifestations of prejudice		1

### Psychiatric morbidity

Out of the 65 articles, three articles described psychiatric morbidity in general among leprosy-affected persons. Psychiatric morbidity, or mental ill health, was measured with the general health questionnaire (GHQ). The GHQ is a tool for screening and identifying minor psychiatric conditions among adults (GL Assessment, n.d.). There are various versions of the GHQ: GHQ-12, GHQ-28, GHQ-30 and GHQ-60. For the purpose of identifying psychiatric morbidity, the shorter test (GHQ-12) is just as effective as the longer tests (GHQ-28 and GHQ-30) (Goldberg *et al*., 1997). However, the longer tests are more suitable for thorough examinations (GL Assessment, n.d.).

All GHQ-based studies identified that leprosy-affected persons scored highly on the GHQ, and thus are likely to have a high prevalence of psychiatric morbidity. Bharath *et al*. (2001) found that the mean GHQ-12 score of the leprosy patients in Bangalore, India was 5.43. Scores of 2 and higher are associated with psychiatric morbidity. Sanyal *et al*. (2011) reported that more than the half of the leprosy-affected persons studied at a leprosy clinic in Kolkata, India, tested positive for psychiatric morbidity on the GHQ-60 (Sanyal *et al*., 2011).

Moreover, Bhatia *et al*. (2006) compared the presence of psychiatric morbidity, measured with the GHQ-12, among leprosy-affected persons and healthy persons from Delhi, India. They found that leprosy-affected persons had a significantly higher prevalence of psychiatric morbidity (44.4%) than healthy persons (7.5%).

#### Depressive disorders

The most frequently identified psychiatric condition among leprosy patients is depression. Of the 27 articles that reported depression in combination with leprosy, the majority reported on the prevalence of depression. The prevalence of depression varied in different settings and countries. For example, a study from Kumar and Verghese (1980) investigated depression among almost 500 leprosy patients from Gudiyattam, India, and found a prevalence of 8.1%, whereas a study executed by Kisivuli *et al*. (2005) among 152 leprosy-affected persons in West Kenya found a prevalence of 49.4%. Weiss *et al*. (1992) investigated the prevalence of depressive disorders among 56 leprosy patients from Mumbai who had recently started treatment and found a prevalence rate of 71%.

Additionally, several studies reported the severity or specific form of depression that was found among leprosy-affected persons. For instance, a recent study conducted by Moura *et al*. (2017) researched the severity of depression among leprosy-affected persons in a referral hospital in Minas Gerais, Brazil. Out of 56 people, 19 had mild depression (34%), 9 had moderate depression (16%) and 5 had severe depression (8.9%), measured with the Hamilton Rating Scale for Depression. Also, Jindal *et al*. (2013) looked at types of depression. They found that among 133 Punjabi leprosy patients, 25.5% suffered from dysthymia, 15% had experienced a moderate depressive episode and 3% had experienced a mixed anxiety and depressive episode.

Moreover, a few studies compared prevalence of depression in leprosy-affected persons with that among healthy persons. Erinfolami and Adeyemi (2009) found that the prevalence among Nigerian leprosy-affected persons was significantly higher than in the healthy controls (respectively 35.2 and 8%). Studies by Nishida *et al*. (2006) and Tsutsumi *et al*. (2004) also confirmed this result in their studies.

#### Anxiety disorders

Anxiety disorders were the second most frequently reported mental health condition, highlighted in 17 out of 65 articles. The prevalence of anxiety disorders among leprosy-affected persons ranged from 10% to 20%. The studies reported different types of anxiety disorders. For instance, Mahendra *et al*. (2016) found that of the 22.7% leprosy-affected persons studied in Bareilly, 11.4% suffered from generalised anxiety disorder (GAD), 4.5% from panic disorder, 4.5% from mixed and other anxiety disorders and 2.3% from obsessive compulsive disorder (OCD). In addition to GAD, OCD and panic disorder, Rocha-Leite *et al*. (2014) also identified agoraphobia (11.7%) and social phobia (9.2%) among leprosy patients in Salvador, Brazil. In addition, a few studies identified mixed anxiety and depressive disorders among leprosy-affected persons (Bhatia *et al*., 2006; Jindal *et al*., 2013; Bakare *et al*., 2015). Erinfolami and Adeyemi (2009) found that Nigerian leprosy-affected persons studied had a significantly higher prevalence of anxiety than healthy persons, respectively 21.6 and 6.8%.

#### Suicide

Suicide, suicide attempts and suicidal thoughts are also common in leprosy-affected persons. Nine studies researched suicide (attempts and thoughts) among this group. According to Bharath *et al*. (1997), 33.3% of the investigated leprosy patients in Bangalore, India dealt with suicidal thoughts, and 20% had attempted suicide. Nishida *et al*. (2006) investigated suicides among elderly leprosy-affected persons in a Japanese leprosarium over a period of almost 100 years and found that in that period, 41 suicides were reported. In a retrospective study by Shen *et al*. (2011), the deaths of 524 Chinese leprosy-affected persons were investigated. Sixteen percent of the cases committed suicide.

#### Schizophrenia

Another mental condition that was found among leprosy-affected persons is schizophrenia. This was found in five studies. However, the prevalence of schizophrenia was low: around 1%. Mahendra *et al*. (2016) found a prevalence of schizophrenia of 2%, but combined schizophrenia together with delusional disorder.

#### Mental distress

Three studies looked at mental distress among leprosy-affected persons (Leekassa *et al*., 2004; Damte *et al*., 2011; Reis *et al*., 2014). Two large studies among Ethiopian leprosy patients found a high prevalence of mental distress: 52.4 and 30.9% (Leekassa *et al*., 2004; Damte *et al*., 2011). Another study among Brazilian leprosy-affected persons identified a prevalence of 48.5% (Reis *et al*., 2014). In addition, Leekassa, Bizuneh and Alem (2004) found that the prevalence of mental distress among Ethiopian people affected with leprosy is 12 times higher than in healthy people (Leekassa *et al*., 2004).

#### Other neuropsychiatric conditions

Investigators also described other conditions linked to leprosy including sleep disorder, dementia, hysteria, epilepsy, paranoid and psychotic state, senile degeneration, delusional disorder, hypochondriacal disorder, adjustment disorders, substance-related disorders, somatic symptom disorder, somatoform disorders, hyperactivity disorder and premenstrual dysphoric disorder. (Miyeko, 1962; Kumar and Verghese, 1980; Nishida *et al*., 2006; Su *et al*., 2012; Rocha-Leite *et al*., 2014; Nagargoje *et al*., 2015; Mahendra *et al*., 2016). Hypochondriacal disorder and dementia were the only conditions that were mentioned in more than one study. However, the prevalence of hypochondriacal disorder was low in both studies (1%) (Rocha-Leite *et al*., 2014; Mahendra *et al*., 2016). In addition, two Japanese studies and one Taiwanese study found dementia among the leprosy-affected persons studied (Miyeko, 1962; Nishida *et al*., 2006; Su *et al*., 2012). The Taiwanese study found a high prevalence of dementia (45.7–50.4%) among leprosy patients (Su *et al*., 2012). However, it should be noted that all three studies were executed among elderly leprosy patients and no comparison was made with elderly people without leprosy.

### Negative feelings and attitudes

In addition to mental health conditions that are diagnosable, other negative feelings and attitudes that can affect mental wellbeing were identified among leprosy patients in almost one-third of the articles.

#### Fear

One of the most frequent negative emotions leprosy-affected persons experienced was fear. A Brazilian study among 130 leprosy patients revealed that patients experience real fear (31.5%) and phantasmal fear (22.3%) (Cunha *et al*., 2015). This fear can originate from different sources related to the disease or related to the person's environment. A study by Garbin *et al*. (2015) revealed that disease-related fear consisted of fear of disease sequelae (92.6%) and fear of dying (27.7%). In addition, Kaur and Ramesh (1994), who studied female leprosy patients in Delhi, found that their patients were also afraid of receiving confronting questions about the disease (77.4%) and of infecting their family members (50%). Even when there was no judgement from family members, the fear of infecting or otherwise negatively affecting them remains among leprosy-affected persons (Anjum *et al*., 2017). Environment-related fear consists of fear of rejection, isolation, stigmatisation and prejudice (Kaur and Ramesh, 1994; Garbin *et al*., 2015; Thilakavathi *et al*., 2015; Zambon Valério Pelizzari *et al*., 2016).

#### Shame

Shame is also very common among leprosy-affected persons, mainly as leprosy causes physical disfigurements. Shame and embarrassment are related to the stigmatisation of leprosy (Van Brakel *et al*., 2012). To illustrate, it is often thought that leprosy is the result of wrong-doing or bad karma (Kaur and Ramesh, 1994; Zambon Valério Pelizzari *et al*., 2016). In addition, leprosy-affected persons often perceive that others are afraid of contracting leprosy. As a result, they avoid talking about their disease or try to conceal it. One of the participants in the study of Zambon Valério Pelizzari *et al*. (2016) explained: ‘*I am embarrassed, friends look and keep asking: What do you have? What did you do to your foot? I am ashamed to tell. I say it was an allergy.*’ In addition, that same study revealed that even medical professionals advised leprosy patients to not discuss their disease with others (Zambon Valério Pelizzari *et al*., 2016). Avoidance of social gatherings can be a result of shame leading to social isolation (Anjum *et al*., 2017).

#### Low self-esteem

As a result of the negative feelings and attitudes towards leprosy, leprosy-affected persons also experience low self-esteem. For instance, they believe that people may avoid people affected by leprosy, or that people may have less respect for them because of the disease, which results in insecurity (Hossain *et al*., 2016). Other researchers showed that discrimination and stigma are also related to low self-esteem (Beise *et al*., 2012; Peters *et al*., 2014). Cunha *et al*. (2015) found that almost one-third of the participants from Minas Gerais, Brazil, experienced low self-esteem. In addition, Reis *et al*. (2014) found that over 60% of the leprosy-affected persons from Rio de Janeiro, Brazil studied lost confidence in themselves. The low self-esteem of patients manifests in, for example, not being willing to talk about the disease in public, isolation from family members and society, and self-stigma (Arole *et al*., 2002; Peters *et al*., 2014; Anjum *et al*., 2017).

#### Loneliness, sadness and anger

A high intensity of emotions like loneliness, sadness and anger was also noticed in leprosy-affected persons. According to Kaur and Ramesh (1994), 70% of the women with leprosy in their Delhi-based study experienced sadness after the diagnosis. Also, 62% revealed that they become angry over irrelevant matters (Kaur and Ramesh, 1994). Loneliness was also found to be present in leprosy-affected persons. A study of Nigerian leprosy-affected persons found that all research participants experienced loneliness (Enwereji, 2015).

#### Low quality of life

Several studies reported a significantly lower quality of life (QoL) for persons affected by leprosy as compared with healthy controls. When leprosy patients from Mato Grosso, Brazil, were asked to rate their QoL in a study by Garbin *et al*. (2015), 37% scored it as ‘very bad.’ Joseph and Rao (1999) found that South-Indian leprosy-affected persons scored significantly lower than healthy controls in the domains of physical and psychological QoL, level of independence, social relationships and environmental QoL. Low scores on the physical and psychological QoL domains were also found in a study in Bangladesh (Tsutsumi *et al*., 2007). Brouwers *et al*. (2011) found that in Nepal, being affected by leprosy resulted in a decreased level of social participation and activity as compared to community controls, in addition to other negative impacts on QoL.

### Mental health of family members

Three studies described the mental health impact of leprosy on family members of leprosy-affected persons. All three studied children and adolescents whose parents were coping with leprosy. In a study by Parashar and Kumar (2015), 100 Indian children of leprosy patients (mean age = 13.6) from Raipur were assessed regarding their physical and psychological health, social relationships and environment. Sixty percent of the children reported having low self-esteem, and none of them reported high self-esteem. In addition, this study found that the children often worried about contracting the disease (Parashar and Kumar, 2015). Another study performed among 102 Nepali children of leprosy patients (mean age = 13.8) compared their health-related QoL, self-esteem and mental health with that of children of healthy controls (Yamaguchi *et al*., 2013). They found that the former group scored higher on depressive symptoms, and lower on health-related QoL and self-esteem. The negative impact of having parents affected by leprosy was also visible in an Indian study by Antony and Broota (1991). They looked at the self-concept of children in Delhi, meaning the view of the child on his/her personality, ability and status in the world. They found that 76% of the children had a negative self-concept.

### Determinants influencing mental health conditions

There were several determinants presented in the articles that can have an influence on mental wellbeing. In the following paragraphs these determinants will be discussed.

#### Stigma and discrimination

Stigma and discrimination were also frequently mentioned factors associated with psychiatric morbidity. Discrimination can result in unemployment, social and marital restrictions, low self-esteem, stress and self-stigmatisation (Beise *et al*., 2012; Van Brakel *et al*., 2012). Stigma can harm the quality of life and social participation of persons affected, and increase mental distress, depression and fear (Bharath *et al*., 1997; Brouwers *et al*., 2011; Damte *et al*., 2011; Thwaites *et al*., 2014; Sillo *et al*., 2016). According to a study among over thousand leprosy-affected persons in Indonesia, 36% experience anticipated stigma (Van Brakel *et al*., 2012). Sillo *et al*. (2016) described the influence of stigma on mental health in their study. They state that physical deformities are the main cause of stigma and disability. Because of the stigma attached to physical deformities, patients experience fear, avoidance, discrimination and prejudice. This can result in diminished mental health, for instance by causing depression and low self-esteem. On the other hand, when deformities cause functional impairments, leprosy-affected persons can become unemployed. Without employment, a decrease in social participation occurs, resulting again in diminished mental health (Sillo *et al*., 2016).

In addition, several case studies revealed that stigma was often mentioned as a reason to attempt suicide (Chatterjee *et al*., 1989; Jopling, 1991). To illustrate this, in the study by Jopling (1991), an Indian leprosy-affected person living in England stated that he would have committed suicide if he had been diagnosed in India, because of the stigmatisation of leprosy in his own country. In addition, 33% of the leprosy-affected persons in the study of Chatterjee *et al*. (1989) admitted having suicidal thoughts because of social stigma.

#### Demographics

Several studies reported that they did not find a relation between mental wellbeing in people affected by leprosy and demographic factors, including age, gender, marital status, educational level, employment or housing (Bharath *et al*., 2001; Erinfolami and Adeyemi, 2009). However, multiple other studies did find an association between demographic factors and mental health, as delineated below.

*Age*. Bakare *et al*. (2015) and Chatterjee *et al*. (1989) found that older age was related to mental wellbeing, specifically an older age at the onset of the disease. Damte *et al*. (2011) found similar results in a study regarding mental distress in Ethiopian persons affected by leprosy. Study results showed that patients in the age group of 48 to 57 had four times more mental distress than patients in a younger age group (18 to 27) (Damte *et al*., 2011).

*Gender*. Two studies found that gender also influences mental wellbeing. According to Joseph and Rao (1999) and Nagargoje *et al*. (2015), female leprosy-affected persons in India had a significantly higher prevalence of psychiatric co-morbidity and a lower quality of life, in all domains, in comparison with male patients.

*Marital status*. Marital status of the leprosy patient can also have an influence on mental wellbeing. Separated or divorced leprosy-affected persons had a four times higher risk of mental distress than single persons, according to one study (Damte *et al*., 2011). Similarly, another study argues that having no spouse can also negatively influence mental wellbeing (Bakare *et al*., 2015).

*Occupation, education and financial means*. Occupation, education and financial means were also found to be associated with mental health status (Bharath *et al*., 2001; Tsutsumi *et al*., 2007; Bakare *et al*., 2015). A study by Nagargoje *et al*. (2015) in Amravati, India, reported that leprosy-affected persons who were employed had a lower prevalence of psychiatric morbidity (71%) than those who were unemployed (90%). In addition, the same study found that illiterate leprosy patients (90%) experienced more psychiatric morbidity than literate patients (66.7%). Moreover, having a lower education level or lower income per year than other leprosy-affected persons was associated with a lower QoL (Tsutsumi *et al*., 2007). According to Thwaites *et al*. (2014), unemployment was also a source of anxiety among leprosy-affected persons.

*Environment*. The environment was another demographic factor associated with mental health status. This was associated with QoL and social participation of leprosy-affected persons (Brouwers *et al*., 2011). Chatterjee *et al*. (1989) studied hospitalised patients and outpatients in Kolkata and Purilia, India. They found that hospitalised patients had a significantly higher rate of mental illness (65%) than outpatients (25%). Enwereji (2015) found similar results, as that study found no depression among discharged leprosy patients. Additionally, Verma and Gautam (1994b) compared leprosy-affected persons from Jaipur, India, living in an ashram with leprosy-affected persons living in a slum. They found that the prevalence of psychiatric morbidity was significantly higher among those living in the slums (Verma and Gautam, 1994b). However, a review found that people who are isolated from the community in ashrams and leprosy homes have a higher risk of developing mental illnesses (Singh, 2012).

Antony and Broota (1991) also looked at the housing situation of the children of leprosy patients from Delhi. They found that 60% of the children living with their parents had a negative self-concept, in comparison with 85% of the children living in institutions for children of leprosy-affected parents.

*Religion*. No association was found between religion and mental health in the included studies.

#### Disease-related factors

*Visible impairments*. A significant link between disease-related factors and mental illness is visible impairments, and worries about developing them. Multiple studies have identified that having visible deformities increases the likelihood of mental illness and negative feelings among leprosy-affected persons (Kumar and Verghese, 1980; Verma and Gautam, 1994a; Bharath *et al*., 2001; Kisivuli *et al*., 2005). For instance, according to a study by Thwaites *et al*. (2014), the possibility of having complications such as blindness, ulcers and contractures was the predominant source of anxiety. Also, studies showed that persons with physical impairments have an increased risk of mental distress and low quality of life (Verghese *et al*., 1971; Leekassa *et al*., 2004; Tsutsumi *et al*., 2007; Damte *et al*., 2011; Parashar and Kumar, 2015). To illustrate this, Verma and Gautam (1994a) found that the prevalence of psychiatric morbidity among persons with visible impairments was 90%, while among those without deformities it was 47%. It is interesting that two studies found that impairments among males affected by leprosy are more frequently associated with mental illness than among females affected by leprosy (Joseph and Rao, 1999; Tsutsumi *et al*., 2007).

In addition, several studies found that patients with multibacillary leprosy experienced mental illnesses more often than patients with paucibacillary leprosy (Leekassa *et al*., 2004; Nagargoje *et al*., 2015).

*Duration of disease*. Another factor that might be associated with the risk of mental illness is the duration of the disease. Some studies argue that the duration of the illness does not affect the mental wellbeing of the person (Verghese *et al*., 1971; Verma and Gautam, 1994a; Bharath *et al*., 2001; Kisivuli *et al*., 2005). However, others did find evidence in their study for an association between the duration of the illness and poor mental health (Kumar and Verghese, 1980; Bakare *et al*., 2015; Nagargoje *et al*., 2015). For instance, Thwaites *et al*. (2014) mentioned that the perception of incurability and re-occurrence of leprosy induces anxiety among people affected.

*Treatment*. The treatment of leprosy patients can also be a factor related to the development of mental illness. Nagargoje *et al*. (2015) found that patients receiving treatment experience more psychiatric morbidity than patients who have completed treatment. The side effects of medications can cause anxiety among affected people (Thwaites *et al*., 2014). The influence of treatment on mental wellbeing was also found in a retrospective study by Shen *et al*. (2011). They aimed to find out whether there was a relationship between the start of multi-drug-therapy (MDT) and suicide. By analysing the deaths of over 500 leprosy-affected persons from China, they found that shortly after the start of MDT, patients had an increased rate of suicide. In addition, the suicide rates also rose steeply after 6 months of using MDT (Shen *et al*., 2011).

Moreover, drugs in the steroid class are also frequently used in the treatment of leprosy-affected persons (Lockwood, 2000). The use of steroids is also related to psychiatric morbidity, for instance suicide and psychosis (Ross and Cetas, 2012).

#### Lifestyle and other factors

Kataoka and Nakamura (2005), who studied elderly persons affected by leprosy in Japan, concluded that higher GHQ scores were found among patients with no or a weak social network, and among patients with an inactive lifestyle. In addition, a study in Nepal found that participation limitations, along with inactivity, negatively influenced the quality of life (Brouwers *et al*., 2011). Social, emotional and health adjustment were also related to poor mental wellbeing in a study in India (Bharath *et al*., 2001). In a study among children between 11 and 18 years old from Agra, India who suffered from leprosy, Chauhan and Dhar (1981) found that anxiety was high among these children because of conflicts with family members, for instance thwarted attempts to gain affection from parents.

### Interventions

In total, 11 interventions were described in the papers identified in the literature search (see Table 4). Interventions ranged from psychological counselling using techniques such as cognitive behavioural therapy (CBT), to physical interventions, for instance surgical correction for the disfigured limbs of leprosy-affected persons. All interventions focused on improving self-esteem and self-worth and on motivating and enabling leprosy-affected persons to participate in society.

#### Psychological interventions

Studies showed that psychological interventions can improve the mental wellbeing of leprosy-affected persons. To illustrate this, the effect of therapeutic workshops to improve the rehabilitation of Brazilian leprosy-affected persons living in a leprosarium was studied by Leite and Caldeira (2015). The therapeutic workshops consisted of creating an environment where leprosy-affected persons could share experiences, socialise and collectively work on problems encountered. By analysing depression and QoL scores, they found that therapeutic workshops lowered the depression prevalence among Brazilians affected by leprosy from 79% to 46.8% and improved their quality of life significantly (Leite and Caldeira, 2015).

Another form of psychological intervention that has been studied is CBT. Directed Imagination, a type of CBT in which desirable behaviour is promoted by training the imagination, was offered to 24 Mexican leprosy-affected persons [Pérez–Hernández *et al*. (2015)]. This therapy improved the adaptation level of the participants from 92% to 96%. In another study by Rahmawati and Yuniarti (2017) CBT was used with 25 leprosy-affected persons dealing with depression. Study results showed that after the intervention depression rates lowered with 12% (Rahmawati and Yuniarti, 2017). Parashar et al. (2015) used a similar technique called Directed Imagination.

A study by Su *et al*. (2012) investigated the effect of reminiscence group therapy (RGT) on depression rates among elderly leprosy-affected persons in Taiwan. They found that the RGT significantly lowered depression scores as measured with the Geriatric Depression Scale – Short Form (Su *et al*., 2011).

#### Physical interventions

Regarding physical interventions, Ramanathan *et al*. (1991) studied the impact of surgical correction of 25 leprosy-affected persons with visible impairments, with the aim to improve their functional, occupational and economic status in society. According to this study, surgical correction diminishes anxiety levels by 24% and depression rates by 32% (Ramanathan *et al*., 1991). Another study combined physical with psychological interventions in institutionalised leprosy-affected persons. Mhasawade (1983) investigated the effect of psychotherapy in combination with drug therapy on depression and anxiety rates among 120 Indian leprosy-affected persons. After psycho- and drug therapy, anxiety and depression rates were significantly lowered (Mhasawade, 1983).

### Model of the impact of leprosy on the mental health

**Fig. 2.**
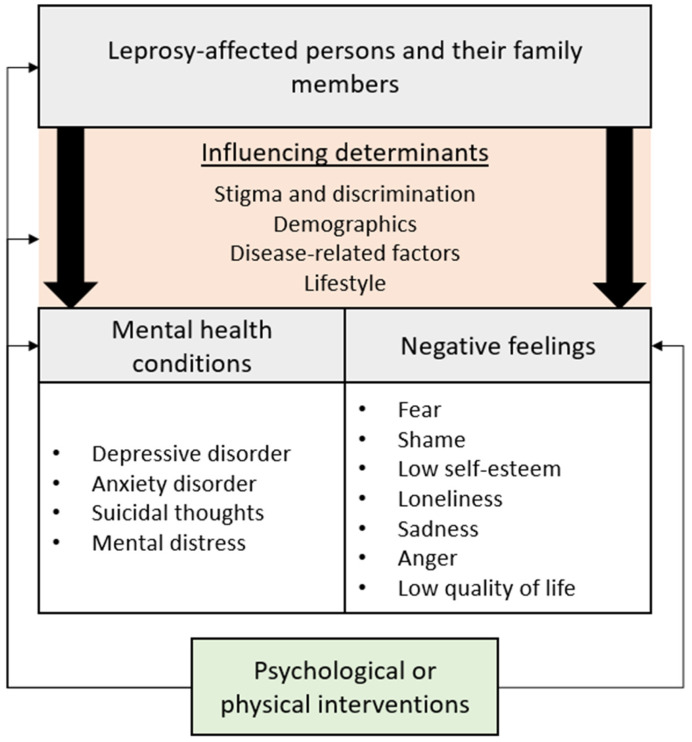
Model of the impact of leprosy on the mental health.

The results of this study can be captured in a model (Fig. 2). This model shows the impact of leprosy on the mental health of those affected and their family members as found in literature. In addition, the influencing determinants are included. As stated in the previous section, there are promising psychological and physical interventions that target mental health conditions, negative feelings or the influencing determinants. These interventions can improve the lives of leprosy-affected persons and their family members.

## Discussion

This study investigated the impact of leprosy on the mental health of leprosy-affected persons and their family members by conducting a systematic literature review. Several psychiatric conditions were reported to occur frequently among leprosy-affected persons, specifically depression (up to 71%), suicide (attempts) (around 33%) and anxiety disorders (10 to 20%). In addition, this study found that children of leprosy-affected persons also experience poor mental health.

Leprosy has physical and psychological consequences that can lead to activity limitations, economic and physical dependence, social exclusion and stigma. All these factors are correlated and can worsen the mental wellbeing of the leprosy-affected persons (Bainson and Van den Borne, 1998; Van Brakel *et al*., 2012). Twelve out of 65 studies included in this systematic review compared the mental consequences of leprosy with either healthy controls or other patients with (chronic) diseases. Most of these studies showed that leprosy has a strong impact on mental health. For example, Bharath *et al*. (1997) compared psychiatric morbidity among patients with leprosy and patients with psoriasis in Bangalore, India. They found a significant difference between the two groups in the prevalence of psychiatric morbidity (12.2% for patients with psoriasis and 47.5% for patients with leprosy). However, more population-based studies are needed to draw conclusions regarding the mental impact of leprosy in comparison with other people, especially healthy populations. Additionally, poor mental health itself can have a negative impact on health outcomes, and may lead to low treatment seeking behaviour and treatment adherence, unemployment and work absence, social exclusion and stigma (Litt *et al*., 2012; Corrigan *et al*., 2014; Banerjee *et al*., 2017). Also, in children and adolescents the negative effects of poor mental health are visible. According to Fergusson and Woodward (2002), adolescents coping with depression experience long-term complications regarding employment, education and health. Experiencing both leprosy and poor mental health is thus a heavy burden to carry for those affected and their family members.

Therefore, a proper evaluation of the burden of leprosy should take into account the psychological and social impact of the disease, as well as the chronic nature of many of its consequences. Incorporating the mental health burden of leprosy with the impact of physical impairments would give a different picture of the overall burden of the disease. Recent studies have attempted to do this for other NTDs with a long-term impact and an underestimation of the DALYs, for instance for leishmaniasis (Bailey *et al*., 2017). Other measures to calculate the disability burden could also be considered, for instance, the SALSA Scale, a measure to estimate the level of activity and safety awareness, or the Participation Scale can be used to gain in-depth insights into the impact of leprosy (Van Brakel and Officer, 2008; Richardus, 2013). A study by van ‘t Noordende *et al*. (2016) reviewed tools to assess and monitor NTDs including leprosy. They found that the Participation Scale was a suitable measure to assess the level of participation, as leprosy-affected persons often cope with participation restrictions. Another alternative measure that can be used to assess the burden of leprosy is the DAWLY. The DAWLY includes the number of productive years lost due to disability and takes the economic consequences of leprosy into account (Rao *et al*., 2013).

There are certain strengths and limitations to this study. First, this study provides a comprehensive overview of the mental burden of leprosy. In addition, other prominent negative feelings among leprosy-affected persons are presented, along with the mental burden on family members, determinants influencing the mental health burden and promising interventions to relieve the burden. A limitation is that, although multiple online databases have been searched, including databases specific for leprosy and NTDs, our search did not include databases specifically targeted at low and middle-income countries.

The findings of this study should be interpreted in the light of certain issues that may influence the occurrence and prevalence of mental health conditions among leprosy-affected persons. First, diagnosing a mental health condition is a complex process and is dependent on various factors. For instance, there is often a spectrum of illness that a person can fit into: depression can range from mild and transient episodes to severe depression with psychosis. In addition, there are many disorders that cannot be precisely diagnosed and that are categorised within ‘somatoform disorder’ (Mayou *et al*., 2005). There are also cultural or language-related aspects of diagnosing mental conditions that relate to diagnostic tools, disease presentation and stigma. Diagnostic and screening tools are not always culturally or semantically appropriate. It is important that these tools are culturally validated before use in a new language or setting. Moreover, cultural factors may impact disease presentation. For example, in some cultures persons experiencing mental distress are more likely to present with physical complaints than to discuss emotions (Stevelink and van Brakel, 2013). In addition, the diagnostic classification of mental disorders can also provoke stigmatisation, as people having a mental illness are marked as different from others in society (Corrigan, 2007). Second, it is possible that the mental disorders found among leprosy-affected persons are not manifesting because of leprosy, but because of other factors. The studies reviewed here often used univariate analysis and were thus not able to take confounding factors into account. For example, Verghese *et al*. (1971) found an association between leprosy and neuroticism. According this study, leprosy patients share traits such as timidity, dependency and impulsivity with people who have confirmed neuroticism. However, these results can also be linked to living in a leprosarium. Another example is that leprosy is closely associated with extreme poverty, which is itself a risk factor for mental ill health (World Health Organisation, n.d.). In addition, study characteristics such as the setting, design or population size can also influence the occurrence and prevalence of mental conditions among leprosy-affected persons.

Also, the prevalence of certain disorders among leprosy-affected persons should be interpreted with caution in studies that did not include an adequate control group, as they could also be similar to the prevalence found in the general population, as is the case for schizophrenia (McGrath *et al*., 2008). Our review shows that leprosy is likely to have a substantial impact on mental wellbeing. Policy makers and healthcare providers should therefore give careful attention to measures to improve the mental wellbeing of those affected. If the mental health impact is taken into account when calculating the burden of disease associated with leprosy, a much more accurate picture can be communicated and the need for interventions to relieve the burden on leprosy-affected persons would be more evident. In addition, further population-based studies to document the prevalence of common mental health consequences such as depression and anxiety among persons affected by leprosy would help greatly to highlight the need for interventions. Several promising interventions to strengthen the mental wellbeing of those affected by leprosy, such as CBT were identified in this study. However, to assess the effectiveness of these interventions on a larger scale, more evidence is needed.

In addition, this study found that leprosy also has a great mental impact on the children of leprosy-affected persons. Further investigation is essential to explore the impact of leprosy on other family members, and to identify suitable interventions to mitigate any negative impact. Multivariate analysis is needed to determine the relation between other known determinants of mental wellbeing, leprosy and mental health. Identifying which determinants play a role in the mental wellbeing of leprosy-affected persons can help to establish suitable interventions. Further investigation of the burden of leprosy on mental wellbeing, should identify the determinants involved, based on which suitable interventions directed at strengthening the resilience and mental health of leprosy-affected persons can be formulated. Additionally, the quality of several studies was poor or could not be assessed due to the limited information provided. We recommend that large, well-designed studies be done to provide data on mental condition in persons affected by leprosy and on the effectiveness of various interventions. These should be reported according to state-of-the-art protocols.

### Limitations

This systematic review focused on a selected number of electronic search engines. However, one of these, Infolep, contains almost all known publications on leprosy, including grey literature. We are therefore confident that no major studies were missed, except if they were published in a language not included in this review and did not contain at least an English abstract. The search strategy was not specifically designed to find intervention studies, but since the overall number of studies on this topic is very limited, we are confident that the studies included give a complete overview of the possibilities regarding interventions. The information contained in quite a few studies was insufficient to rate the quality of the studies objectively. However, given the limited number of studies available on this topic overall, we decided not to exclude these from the review.

## Conclusion

This is the first study to consolidate the evidence of the mental health impact of leprosy on affected persons and their family members through a systematic literature review. A number of mental health disorders and negative emotional states were identified among leprosy patients and their family members. Depression and anxiety are particularly common, and a higher prevalence was found among leprosy-affected persons in comparison with healthy controls. Low self-esteem and low QoL were found among those affected by leprosy and the children of leprosy-affected persons. Additionally, determinants were identified that may influence mental wellbeing other than leprosy itself, especially stigma and discrimination and (visible) impairments, but also demographic factors and other disease-specific factors. Further research is necessary to identify the burden of disease of leprosy, taking the mental health consequences into account, and the impact of leprosy on the mental wellbeing of family members. In order to prevent and mitigate the mental health impact of leprosy, interventions are needed to strengthen coping mechanisms of those affected, to treat mental health conditions such as depression in this population, and to change negative community attitudes towards those affected by the disease, as stigma is a key contributor to mental ill health.
